# Never let a good crisis go to waste: Pauline Hanson’s exploitation of COVID-19 on Facebook

**DOI:** 10.1177/1329878X20953521

**Published:** 2021-02

**Authors:** Kurt Sengul

**Affiliations:** The University of Newcastle, Australia

**Keywords:** communication, COVID-19, Facebook, Pauline Hanson, populism, populist radical right, social media

## Abstract

This brief contribution explores how the 2020 COVID-19 crisis has been exploited by Australian populist radical right politician, Pauline Hanson. In particular, I discuss how Hanson, through her political communication on Facebook, has used the COVID-19 crisis to prosecute her longstanding nativist policies on issues like immigration. I further discuss how Hanson’s anti-Asian and Sinophobic rhetoric has occurred alongside an increase in anti-Asian racism in Australia throughout the COVID-19 crisis. I conclude by discussing the implications of this as well as foreshadowing future empirical work on this topic.

The unprecedented scale of the COVID-19 crisis has necessitated an enormous global public health and economic response. While much of the focus of the crisis has understandably been on the immediate health and economic implications, the response of the far-right to COVID-19 also requires critical examination. The far-right, and in particular, the *populist radical right* (Mudde, 2007), has historically exploited great crises in order to scapegoat minorities and legitimise a range of exclusionary immigration policies (Mudde, 2019; Wodak, 2015). As such, this essay critically explores how Australia’s most prominent populist radical right politician, Pauline Hanson, has used Facebook to exploit the COVID-19 crisis. It is argued here that through her political communication, Hanson has sought to exploit the COVID-19 crisis by manufacturing anti-Chinese sentiment in Australia with the purpose of legitimising her party’s long-standing policies on issues like immigration. Australia has experienced an increase in racist attacks against Asians and Asian Australians throughout COVID-19 as documented in the *COVID-19 Coronavirus Racism Incident Report* (Chiu and Asian Australian Alliance, 2020), and thus it is important to interrogate the role of influential elite political actors such as Pauline Hanson in promoting this racism.

This essay in particular focuses on Pauline Hanson’s political communication via Facebook as this is the social media platform where Hanson is most prolific, with 340,000 followers – the second largest following of any Australian political leader behind the prime minister Scott Morrison.^1^ Social media sites like Facebook offer the far-right a relatively, albeit not entirely, unmediated platform to communicate directly with their supporters when compared to other forms of communication. As Wodak (2015) argues, the populist radical right has proven to be adept at exploiting the affordances of social media platforms. As noted by Engesser et al. (2017), the features of populist style, ‘such as simplification, emotionalization, and negativity, are perfectly in line with the Internet’s attention economy’ (p. 1286). Pauline Hanson’s use of Facebook has been a key pillar of her political communication strategy. In his typology of populist leaders’ level of social media presence, Moffitt (2019) categorises Hanson as having a s*trong social media presence* alongside other populist actors including Geert Wilders, Rafael Correa and Sarah Palin. Indeed, Hanson’s Facebook page can be described as very active, frequently updated and often includes dialogic engagement with her followers. Hanson’s Facebook following is substantially larger than her Twitter account which has almost 60,000 followers.

Like most of her contemporaries on the populist radical right, Hanson’s 21st century resurgence has largely focused on Muslims as the new ‘dangerous Other’ (Sengul, 2020), with Islamophobia being the defining prejudice of her contemporary political career. However, Hanson’s first iteration has a member of the House of Representatives in the 1990s (1996–1998) largely focused on First Nations people and Asian immigration (see, for example, Grant, 1997; Gray and Winter, 1997), infamously stating in her 1996 maiden speech that ‘I believe we are in danger of being swamped by Asians’ (Hanson, 1996). The populist radical right always constructs an exclusionary nativist form of ‘the people’ who ‘are viewed in contrast to various external ‘Others’, such as ‘immigrants, seen as a threat to the identity and prosperity of the nation’ (Mouffe, 2018: 24). Right-wing populists ‘successfully construct fear and – related to the various real or imagined dangers – propose scapegoats that are blamed for threatening or actually damaging our society’ (Wodak, 2015: 22). These various Others must be excluded from ‘the people’ as they ‘embody alien and threatening cultural values’ (de la Torre, 2015: 11). The COVID-19 crisis has augmented Hanson’s Sinophobic rhetoric through her political communication of Facebook.

A broad consensus exists within the literature that populists tend to be adept at both exploiting and manufacturing crises to their political advantage (see, for example, Moffitt, 2016; Mudde, 2019; Wodak, 2015). As Cas Mudde notes, the contemporary populist radical right have profited politically and electorally from the exploitation of the three most significant 21st century crises: The September 11 Terrorist Attacks, the 2008 Global Financial Crisis and the 2015 so-called ‘refugee crisis’ (Mudde, 2019). The populist radical right has sought to use these crises to scapegoat racialised people, immigrants and minorities, and to legitimise a range of nativist and exclusionary policies.

Throughout the COVID-19 crisis, Hanson’s response has had a particular focus on China and has revived the Sinophobic rhetoric that was typical of her first iteration as a parliamentarian in the late 1990s. Hanson has echoed Donald Trump’s Sinophobic language, almost exclusively referring to COVID-19 as the ‘Chinese virus’ and has sought to prosecute her existing nativist agenda on issues such as immigration, welfare and trade. Unlike her first iteration however, Hanson’s contemporary resurgence occurs within what Mudde (2019) refers to as ‘the fourth wave of the far-right’ (p. 169), whereby the populist radical right has become increasingly tolerated, normalised and mainstreamed in the 21st century. Hanson’s political communication therefore is arguably more consequential in the contemporary context given that her ideas have been increasingly accepted in the mainstream (Mudde, 2019).

Hanson has used Facebook to prosecute a number of populist and far-right demands throughout COVID-19. For example, Hanson has used the crisis to call for Australia to withdraw from its free trade agreement with China, has urged Australians to boycott products made in China, called for the immediate suspension of all Chinese foreign investment into Australia and suggested that backpackers and foreign workers should be denied welfare assistance and called for Australia to cease all foreign aid. Hanson’s populist rhetoric has been most frequently articulated through her attacks on international organisations like the World Health Organization and United Nations (UN). Hanson has railed against ‘corrupt globalist bureaucracies like the United Nations and World Health Organisation . . . [which] act as propaganda arm of the Chinese Government’ and has called for Australia to ‘leave the UN’ and ‘take back our sovereignty’. Further arguing that ‘it’s time to call this out and make sure the United Nations and its left-wing allies can’t use this tragedy to squeeze more money from struggling Australians’. Hanson has also floated the unfounded conspiracy theory that COVID-19 was developed in a Chinese laboratory and has suggested that China has ‘unleashed’ Coronavirus on the world. All of these policies have been long-standing One Nation commitments before the COVID-19 crisis, and it is clear that Hanson is seeking to exploit and manufacture anti-Chinese sentiment to re-prosecute this agenda. For example, one of Hanson’s Facebook posts encouraged followers to boycott the purchase of Chinese made products, arguing that (capitalisation in original):

IF IT’S MADE IN CHINA, LEAVE IT ON THE SHELF. (Hanson, 2020b)

A key discursive tool of the populist radical right, and one that Pauline Hanson frequently uses (see, for example, Sengul, 2019, 2020), is *negative-Other presentation* which refers to the assigning of negative qualities to a particular out-group (Wodak, 2015). This strategy has been prominent during Hanson’s COVID-19 Facebook posts relating to China and serve the function of Othering. Two prominent examples include

CHINESE VULTURES CIRCLE VIRGIN AIRLINES–AUSTRALIA MUST SAY NO. (Hanson, 2020a)

THE SNEAKY CHINESE TRICK DECEIVING AUSSIE SHOPPERS. (Hanson, 2020c)

Hanson’s anti-Chinese rhetoric on Facebook throughout the COVID-19 crisis has occurred alongside an increasing number of racist attacks against Chinese and Asian people living in Australia (Tan, 2020). COVID-19-related racism complaints have made up about a third of the Human Rights Commission’s caseload this year. As Carland (2020) argues, the economic consequences of the COVID-19 crisis are likely to lead to further scapegoating of ethnic minorities and immigrants as people start to look for someone to blame. As an influential and powerful political actor, Hanson’s political rhetoric has consequences. Research has demonstrated that right-wing populist rhetoric on Facebook is consequential. For example, a recent study of right-wing populist communication found that citizens who held strong anti-immigrant views were ‘more likely to selectively expose themselves to RWP [right-wing populist] content, and exposure to such content reinforced their initial anti–immigrant attitudes’ (Heiss and Matthes, 2020: 18). These results, according to the authors, suggest the strategic value and importance of social media channels for right-wing populists in both spreading messages and increasing electoral support for them (Heiss and Matthes, 2020). Similarly, research by Hameleers (2019) has found that anti-immigrant right-wing populist messaging ‘activate negative stereotypical images of migrants among people with stronger perceptions of relative deprivation’ (p. 1). In this context, Hanson’s Sinophobic political communication during the COVID-19 crisis must be understood as consequential in further marginalising racialised people and reproducing racist attitudes.

The purpose of this brief essay was to demonstrate how Pauline Hanson is exploiting the COVID-19 crisis through her communication on Facebook. With the second largest following of any political leader in Australia, Facebook provides Hanson with a significant platform to spread political messages. As the economic and social consequences of COVID-19 manifest, understanding how the populist radical right exploits crises in order to scapegoat marginalised groups becomes increasingly important. It is clear that Pauline Hanson views the COVID-19 crisis as an opportunity to legitimise her nativist views around immigration, and Facebook provides a useful platform to spread these messages. The scale of COVID-19 has required an enormous public health and economic response, but we must also be alert to the impact of the crises on racialised minorities who have historically bore the brunt of scapegoating by the far-right during times of crisis. This debate occurs in the context of Facebook resisting calls to regulate hate speech and the fact-checking of political statements as has recently been adopted by Twitter (Brown, 2020). Indeed, a key recommendation from the *COVID-19 Coronavirus Racism Incident Report* (Chiu and Asian Australian Alliance, 2020) was for social media platforms to make it easier to report and remove trending content that is confirmed as false that may encourage COVID-19-related anti-Asian racism (p. 20). Future empirical research should seek to understand how social media sites like Facebook provide a vehicle for the radical right to produce and reproduce racist rhetoric is increasingly paramount. Moreover, we should examine the specific communicative and discursive strategies that Hanson has been using throughout the COVID-19 crisis to further our understanding of how the far-right exploits and manufactures crises online.

## Notes

1. As of 7 August 2020, Pauline Hanson has 354,000 Facebook followers. The Prime Minister Scott Morrison has 655,000 followers. The leader of the Labour party, Anthony Albanese has 140,000 followers. The leader of the Australian Greens, Adam Bandt has 132,000 followers.
